# Supplementary figures and images for: The Ligurian Experience in the Management of Lung Cancer: Organizational Models and New Perspectives
Source: Healthcare (Basel). 2024 Dec 18;12(24):2556. doi: 10.3390/healthcare12242556 (PMC11675364; doi:10.3390/healthcare12242556)

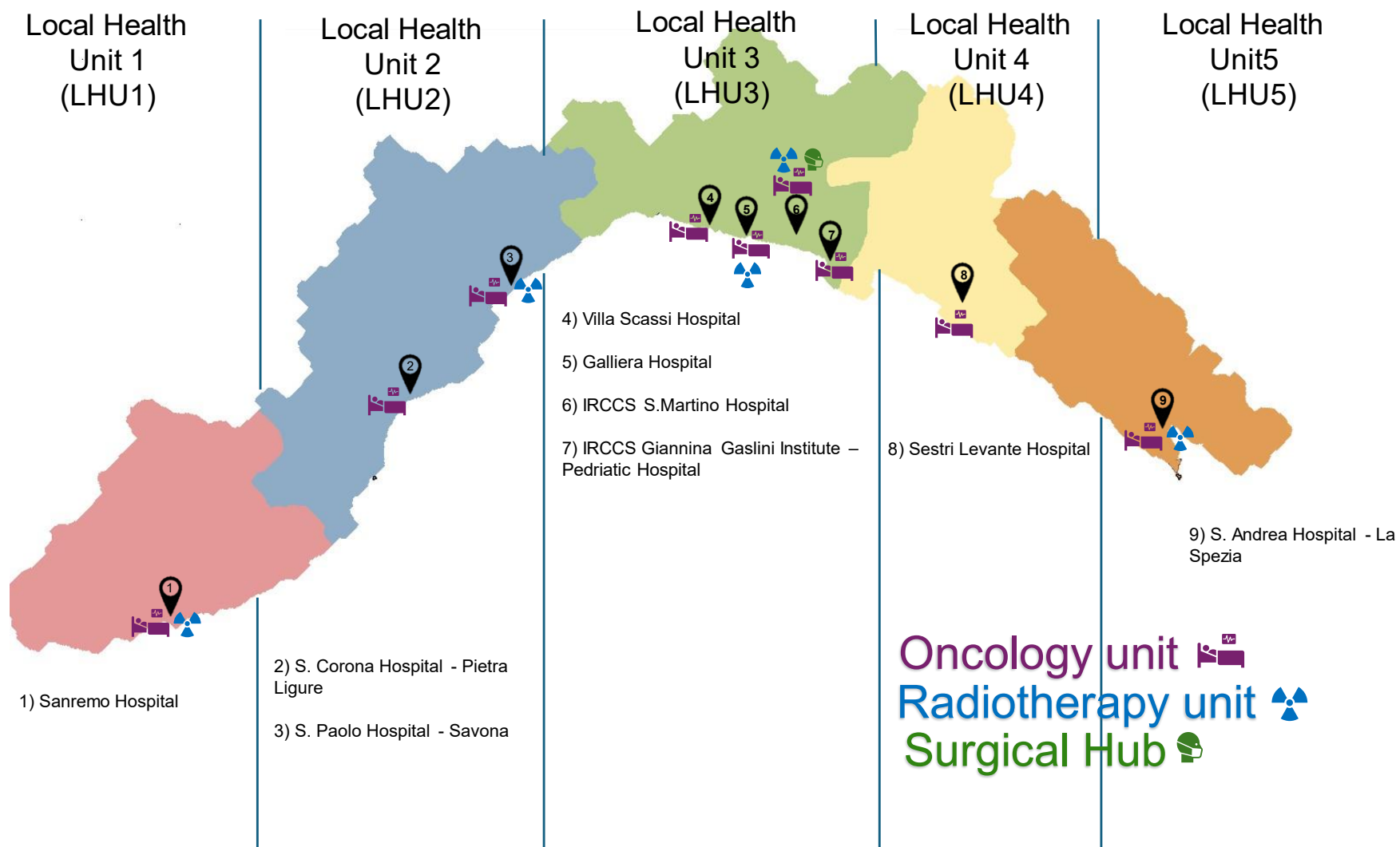

Figure S1: The key units dedicated to the care of oncologic patients.

Supplement: Supplementary file 1 [file healthcare-12-02556-s001.zip › healthcare-3309880-supplementary.pdf]
